# Interplay between P-Glycoprotein Expression and Resistance to Endoplasmic Reticulum Stressors

**DOI:** 10.3390/molecules23020337

**Published:** 2018-02-06

**Authors:** Milan Hano, Lenka Tomášová, Mário Šereš, Lucia Pavlíková, Albert Breier, Zdena Sulová

**Affiliations:** 1 Institute of Molecular Physiology and Genetics, Centre of Bioscience, Slovak Academy of Sciences, Dúbravska cesta 9, 84505 Bratislava, Slovakia; milan.hano@savba.sk (M.H.); mario.seres@savba.sk (M.Š.); lucia.pavlikova@savba.sk (L.P.); 2 Institute of Clinical and Translational Research, Biomedical Research Center, Slovak Academy of Sciences, Dúbravska cesta 9, 84505 Bratislava, Slovakia; lenka.tomasova@savba.sk; 3 Institute of Biochemistry and Microbiology, Faculty of Chemical and Food Technology, Slovak University of Technology, Radlinského 9, 81237 Bratislava, Slovakia

**Keywords:** multidrug resistance, P-glycoprotein, ER stress, unfolded protein response, ERAD, *N*-glycosylation, malignancies

## Abstract

Multidrug resistance (MDR) is a phenotype of cancer cells with reduced sensitivity to a wide range of unrelated drugs. P-glycoprotein (P-gp)—a drug efflux pump (ABCB1 member of the ABC transporter gene family)—is frequently observed to be a molecular cause of MDR. The drug-efflux activity of P-gp is considered as the underlying mechanism of drug resistance against P-gp substrates and results in failure of cancer chemotherapy. Several pathological impulses such as shortages of oxygen and glucose supply, alterations of calcium storage mechanisms and/or processes of protein *N*-glycosylation in the endoplasmic reticulum (ER) leads to ER stress (ERS), characterized by elevation of unfolded protein cell content and activation of the unfolded protein response (UPR). UPR is responsible for modification of protein folding pathways, removal of misfolded proteins by ER associated protein degradation (ERAD) and inhibition of proteosynthesis. However, sustained ERS may result in UPR-mediated cell death. Neoplastic cells could escape from the death pathway induced by ERS by switching UPR into pro survival mechanisms instead of apoptosis. Here, we aimed to present state of the art information about consequences of P-gp expression on mechanisms associated with ERS development and regulation of the ERAD system, particularly focused on advances in ERS-associated therapy of drug resistant malignancies.

## 1. Introduction

**Figure 1:**
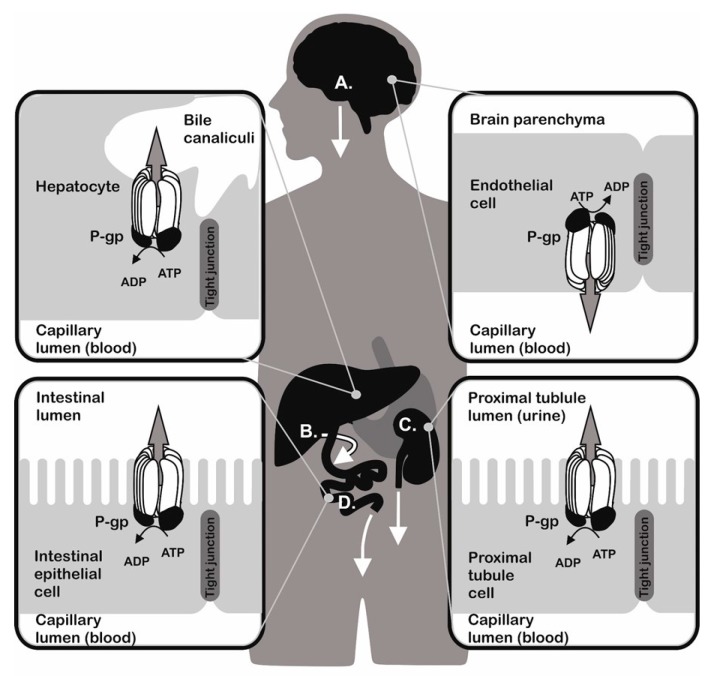
Biological function of P-glycoprotein (P-gp) in various tissues. (**A**) The blood-brain barrier separates blood circulating in the brain capillaries from the brain tissue. P-gp is one of the blood-brain barrier components and is localized on the luminal side of brain micro-capillary endothelial cells, where it prevents the penetration of xenobiotics through the endothelium to brain tissue; (**B**) P-gp promotes the excretion function of liver by transporting substances with limited solubility in water and their metabolites formed by enzymes of the first and second phase of liver detoxification machinery from hepatocytes into the bile; (**C**) P-gp in the proximal tubule cells of the nephrons secures elimination of water soluble substances via urine; (**D**) The gut blood barrier prevents the absorption of pathogens and toxins from diet [21,22]. P-gp is localized in the mucosal cells of the small intestine and promotes the integrity of the gut blood barrier by pumping harmful substances into the lumen of the intestines.

Organisms were persistently exposed to environmental attacks represented by different chemicals during evolution. To survive the unfavourable conditions, they developed functions to detoxify and remove toxins. Effective detoxification mechanisms are present both in unicellular (like bacteria and yeast) and multicellular (like plants and animals) organisms. These mechanisms are responsible for formation of defence systems against a broad spectrum of structurally unrelated substances that induce cell damage by different mechanisms [1,2,3,4,5,6,7,8,9,10]. The ATP Binding Cassette (ABC) transporters were proposed as universal detoxifiers, since they are present in a wide variety of organisms. They either transport substances out of cells or into the lumen of intracellular organelles. P-glycoprotein (P-gp, an ABCB1 member of the ABC transporter family) is encoded by the *MDR1* (*ABCB1*) gene [11] and represents the first discovered [12] and most studied ABC transporter. The tissue distribution of P-gp is linked to its function as a transporter responsible for excretion of different compounds (Figure 1). P-gp is present at a higher amount in: (i) mucosal cells of the small intestine [13]; (ii) in the endothelial cells either of the blood brain barrier [14] or blood placental barrier [15], where it prevents the absorption of toxins; and (iii) in the kidney proximal tubule and hepatocytes, where it pumps metabolites and xenobiotics into the urine (substances that can be dissolved in urine) and bile (substances with limited solubility in water milieu) [16]. The transport function of P-gp protects cells against accumulation of harmful substances and thus plays an important role in maintaining physiological homeostasis. On the other hand, P-gp secured elimination of drugs, particularly anticancer agents, from the inner space of cells leads to the loss of pharmacological responses and development of multidrug resistance (MDR) to P-gp substrates. This phenomenon may result in chemotherapy failure in patients and consequent impairment of therapy outcome [17,18]. Similarly, analogues of P-gp either in bacteria [19] or in protozoa [20] were described to reduce sensitivity to antibiotics and anti-malarial drugs, respectively.

## 2. P-Glycoprotein and Multidrug Resistance (MDR)

Cancer cells can develop specific phenotypes characterized by overexpression of P-gp that confers resistance to a wide range of structurally unrelated substances belonging to a cluster of P-gp substrates. When expressed in neoplastic cells, P-gp can cause massive drug resistance to substrates involving anthracyclines (e.g., doxorubicin), vinca alkaloids (e.g., vincristine), actinomycines (e.g., actinomycin D, dactinomycines), taxols (e.g., paclitaxel), alkylating agents (mitomycin C), peptide antibiotics (gramicidin, valinomycin) and many others (reviewed in [23]). This phenotype could be inherently reflecting special functions of tissue from which neoplastic cells were developed or acquired due to cancer cell selection/adaptation to the presence of anticancer agents [17]. The resistant cells exhibit lower intracellular concentrations of anticancer drugs, which is related to alterations in the plasma membrane, particularly extremely improved drug efflux activity of P-gp that confers resistance to P-gp substrates by several hundred times [2,24]. Upregulation of the *MDR1* gene is found in various cancer types [2,24,25,26,27,28,29,30,31,32]. The drug-efflux activity of P-gp is considered to be the underlying mechanism of MDR [12,33,34]. In addition, direct inhibition of tumour cell apoptosis was proposed in P-gp positive cells [35]. Alteration of apoptosis induced by drugs in P-gp positive cells have been described by several authors [36,37,38,39,40]. This activity is independent on P-gp drug efflux activity since transport-defective mutant P-gp expressed in CEM lymphoma cells suppresses vincristine-induced apoptosis via reduction of mitochondrial cytochrome C release and depressed caspase activation [41]. Moreover, we describe depression of cisplatin sensitivity (a substance that is not a P-gp substrate) in L1210 cells expressing P-gp due to either selection with vincristine or transfection with a human *MDR1* gene [42,43]. P-gp via this antiapoptotic activity could induce significant cell resistance against substances that are not P-gp substrates. 

P-gp is a polypeptide consisting of 1280 amino acid organized in two halves. Both halves have a strong structural similarity and contain a transmembrane domain formed by 6 α-helical membrane spans and an ATP binding site with ABC structural consensus (reviewed in [24]). After the binding of drugs to the intracellular P-gp drug binding domains oriented either to cytosol or inner membrane space, an ATP dependent conformation change of P-gp occurs and the agents are relocated to the extracellular space [44]. P-gp is synthetized on rough ER as a 150 kDa polypeptide precursor, which is after correct folding with calnexin and Hsc70 [45] further glycosylated into a 170 kDa mature protein [46,47]. P-gp moves from the ER to the Golgi apparatus (GA) for glycosylation and is afterwards incorporated into the plasma membrane. The regulation of P-gp trafficking from the ER to the plasma membrane is not completely clear. It was reported that microtubules are required for its transport from ER to GA [48] and a direct or indirect path to the plasma membrane via an intracellular endosome pool has been proposed [49,50]. Disruption of folding or glycosylation of glycoproteins (including P-gp) may lead to rapid proteasome-mediated degradation [51].

## 3. Protein Quality Control in Endoplasmic Reticulum (ER)

**Figure 2:**
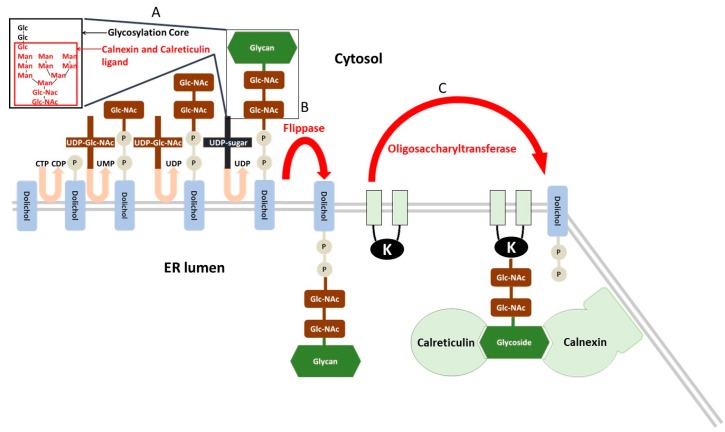
*N*-glycosylation of protein in the endoplasmic reticulum (ER). (**A**) Synthesis of glycosylation core (Glc3Man9NAcGlc2) on dolichol attached to the ER membrane and oriented to the cytosol; (**B**) Relocation of the newly synthetized glycosylation core from the cytosolic to the luminal side of the ER by distributing flippase [58]; (**C**) Transfer of glycosylation core from dolichol phosphate to protein by oligosaccharyltransferase (EC 2.4.1.119) [62] and specific linkage of new glycoprotein with Ca^2+^-dependent lectins/chaperones of ER calnexin and calreticulin [63,64] due to its affinity for the oligosaccharide moiety labelled by a red square in A.

The endoplasmic reticulum (ER) is an organelle that secures cell homeostasis via serving the following functions: (i) proteosynthesis on ribosomes attached to rough ER; (ii) control of protein posttranslational modification, their folding and intracellular translocation; and (iii) storage of cell calcium and regulation of calcium homeostasis. In the case of correct folding, proteins enter the secretory pathway in the ER and GA [52]. *N*-glycosylation is the crucial step in the posttranslational modification in ER and represents a basic protein quality control [53]. The *N*-glycosylation is initiated in the ER while the protein is folded. Further processing of the *N*-glycan is catalysed by specific glycosidases and glycosyltransferases [54,55,56,57]. The elongation of the *N*-glycans and the *O*-glycosylation proceeds in the GA after the folding. First, the glycoside core (Glc_3_Man_9_NAcGlc_2_) linked with a dolichol phosphate anchored in the ER membrane is synthesized on the cytosolic side and flipped to the luminal side of the ER [58]. The glycoside core has a specific structure (documented in Figure 2) with three terminal glucoses [59]. After synthesis, the glycosylation core is relocated to the NH_2_ group of the asparagine residue of proteins undergoing *N*-glycosylation. Before the translocation to the GA, two chaperone proteins, the soluble calreticulin and the membrane bound calnexin, control the state of protein folding [60,61]. These lectins/chaperones exert Ca^2+^-dependent affinity to structure of glycosylation core with the one terminal glucose (GlcMan_9_NAcGlc_2_). Only properly folded proteins can escape from binding with calnexin and calreticulin and exit the ER. 

Exiting the ER is secured by specific elimination of terminal glucoses with α-glucosidase I and II and consequent loss of ligand property for calnexin and calreticulin [59]. Unfolded proteins are reglucosylated by the UDP glucose:glycoprotein glucosyltransferase (UGGT) and re-enter the calnexin/calreticulin cycle several times to complete the folding [52]. However, persistently misfolded proteins are recognized by mannosidases, which remove mannose residues from the glycan [65,66] and thus prevent the reglucosylation by UGGT [67] and further calnexin/calreticulin protein quality control [52]. This is the first step of the ER-associated protein degradation (ERAD). After binding to the ERAD lectins, osteosarcoma amplified 9 (OS-9) or XTP3 transactivated protein (XTP3-B or erlectin), the deglycosylated proteins are removed to the cytoplasm and delivered for ubiquitination [68,69]. The transmembrane ubiquitin E3 ligase proteins link the recognition of misfolded proteins in the ER and the proteasome mediated degradation in the cytosol [70]. In the mammals, Hrd1/Suppressor of Lin-12-like (Sel1L) ligase is one of the ligases responsible for ubiquitination followed by degradation in proteasomes [71]. The Hrd1/Sel1L ligase is part of a complex with several other proteins (Derlin 1–3 proteins, p97 ATPase, VIMP, Herp), which extract and dislocate unfolded proteins from the ER membrane to the cytosol [72,73,74]. 

## 4. ER Stress, Induction and Consequences

Under ER stress (ERS) unfolded proteins accumulate in the lumen of the ER, resulting in activation of the unfolded protein response (UPR). The UPR program plays a crucial role in the regulation of the cell survival/death switch [75]. The primary action of the UPR is the modification of pathways responsible for protein folding, removal of misfolded proteins by ERAD and inhibition of protein synthesis in order to preserve the cell against stress induced by an excess of unfolded proteins [76]. However, in the case of sustained stress, UPR induces cell death [75]. Three ERS sensors control the UPR: inositol-requiring enzyme 1α (IRE1α) [77], pancreatic ER kinase (PERK) [78] and activating transcription factor 6 (ATF6) [79]. The activation of these three sensors under non-stress conditions is inhibited by the immunoglobulin binding protein (BiP), known also as glucose regulated protein 78 (GRP78). This protein is an intracellular chaperone with function either in correct folding of nascent polypeptides in ER or UPR regulation, which may protect cells against apoptosis induced by immature protein accumulation in ER. BiP is also known as a member of the heat-shock protein (HSP) 70 family and is upregulated in cells under oxygen and glucose limitation [80].

Accumulation of the unfolded proteins leads to the binding of the BiP to the unfolded proteins, due to a higher affinity of the BiP to unfolded proteins compared to ERS sensors. The dissociation of BiP from the luminal domains of stress sensors results in homodimerization of both IRE1α and PERK, their trans/auto phosphorylation, the translocation of ATF6 to the GA and subsequent activation [52,75].

The mechanisms of switching between cell survival and cell death under ERS and its regulation is not fully understood. The activated ERS sensors promote expression of both pro survival and pro apoptotic mediators [81]. It is believed that there is a mutual regulation between death-inducing and life-sustaining factors. Particularly in the early stage of ERS, the death inducing stimuli are antagonized by pro survival stimuli. On the contrary, long-lasting stress induces an opposite response, shifting the balance in favour of cell death [75] by the following mechanisms:

- The transcription factor X-box-binding protein (XBP1) regulates protein folding, trafficking and ERAD. After IRE1α induction of specific splicing of XBP1 mRNA, spliced variant sXBP1 enters the nucleus and regulates the expression of downstream products as UPR target genes, such as those encoding chaperones and components of ERAD [82,83,84]. The early pro survival response inhibits the expression of pro apoptotic C/EBP homologous protein (CHOP). However, prolonged ERS is associated with an increase of ROS levels and IRE-1α/XBP1 mediated activation of both CHOP and Bim and inhibition of Bcl-2, which leads to apoptosis [85,86,87,88,89].
- Activation of the PERK pathway leads to phosphorylation of eukaryotic initiation translation factor 2α (eIF2α), which triggers an antioxidant, pro survival response mediated by transcription factor 4 (ATF4). These phosphorylation leads to inhibition of translation [90]. In the case of sustained PERK stimulation, ATF4 signalling pathway induces dephosphorylation of eIF2α and promotes the expression of CHOP to mediate ERS-induced apoptosis [91,92].
- The ERS sensor ATF6 moves after activation from the ER to the GA, where it is cleaved by site 1 and site 2 proteases (S1P and S2P) to generate a cytosolic active transcription factor regulating protein folding and degradation [93].

The repression of the transcription factor E2F1, a direct inhibitor of apoptosis, was proposed as the crucial step in determining the death program [75]. It was reported that the late IRE1α/XBP1 response positively regulates the E2F1 gene and activates ATF6. The combined activity of E2F7 and ATF6 results in downregulation of E2F1 followed by a rapid apoptotic response [94].

ER stress may be pharmacologically induced by substances that alter the proper function of the ER [95]:

- Inhibitors of protein *N*-glycosylation, such as tunicamycin, an inhibitor of the UDP-*N*-acetylglucosamine-dolichol phosphate *N*-acetylglucosamine-1-phosphate transferase, that realize the first step of glycosylation core synthesis (Figure 2).
- Inducers of calcium depletion of ER, such as thapsigargin, that block the calcium pump of this organelle. Lack of calcium content in the ER eliminates proper function of calnexin and calreticulin and induces malfunction of protein quality control in the ER.
- Inhibitors of transport of proteins from the ER to the GA, such as brefeldin A, that additionally induce retrograde protein transport from the GA to the ER. This leads to the accumulation of unfolded proteins in the ER.
- Strong reducing agents, such as DTT, that block disulphide-bond formation and induce ERS within minutes.
- Proteasome inhibitors, such as, MG132 that block ERAD and cause misfolded protein accumulation in the ER.

## 5. ER Stress, Cancer and MDR

Uncontrolled proliferation of cancer cells leads to fast tumour growth associated with a low nutrient and oxygen supply that may induce disruption of cell homeostasis and ERS. Despite sustained activation of stress sensors, malignant cells do not switch to apoptosis. In contrast, they are able to adapt to ERS and deregulate the UPR in favour of cell survival, resulting in tumour development and progression [75,96]. Angiogenesis plays a crucial role in the progression of tumours by developing new vascular networks to supply nutrients and oxygen for malignant tissues [97]. All three sensors, PERK, IRE1α and ATF6, were reported to promote the expression of pro angiogenic vascular endothelial growth factor (VEGF) [98,99,100,101,102]. Moreover, neoplastic cells express increased levels of antioxidative factors as protection against the action of reactive oxygen species (ROS) [103]. In this context, PERK mediates the antioxidative nuclear factor-erythroid 2-related factor 2 (Nrf2) pathway and thus promotes the glutathione mediated buffer capacity of the generated ROS [104]. Similarly, activation of PERK was associated with tumour initiation and expansion by maintaining redox homeostasis and protecting the cancer cells from oxidative DNA damage [105]. Inhibitor of apoptosis-stimulating protein p53 (iASPP), a key inhibitor of this tumour suppressor, facilitates tumour growth by promotion of autophagy dependent on mTOR (mechanistic target of rapamycin) [106]. This p53 inhibitor is an antioxidative factor and drives cancer growth and drug resistance by competing with Nrf2 for keap1 (Kelch-like ECH-associated protein 1) binding [103].

The ERS sensors were suggested to play a role in tumour development. Low glucose levels and hypoxia in cancer cells resulted in activation of PERK and XBP1 and downstream pro survival pathways [107,108,109,110]. Several cancer types overexpress XBP1 and CHOP factors, related to higher cell proliferation and poor patients’ prognosis [111,112,113,114,115,116,117]. A transcriptional XBP1/HIF-1α complex was suggested to promote the tumorigenicity and progression of an aggressive subtype of human breast cancer [118]. Finally, overexpression of XBP1s was reported to play a role in the pathogenesis of multiple myeloma and a poor prognosis [119,120]. Conversely, several reports support the anti-tumour role of ERS, e.g., either better clinical prognosis observed in acute myeloid leukaemia patients with induced sXBP1 mRNA [121] or increased proliferation and malignant transformation after inhibition of the PERK/eIF2α pathway [122,123]. This could reflect the specificity of pro-/anti-tumour actions of ERS in different stages and types of malignant tissues.

Several members of the ERAD program have been proposed to play a role in the development of cancer [70]. Increased expression of Sel1L associated component of ubiquitination ligase was detected in different cancer types, which was related to reduced tumour growth and prolonged overall survival [124,125,126,127]. In contrast, glioblastoma multiform cells with a single nucleotide polymorphism rs12435998 genotype downregulated Sel1L, which was associated with a better prognosis of patients [128]. This polymorphism was proposed as a predictor of glioblastoma survival and response to radio-chemotherapy. It was suggested that ER lectin OS9 (known also as ERLEC2) promotes the tolerance of cancer cells to hypoxia by suppressing transcription of and mediating degradation of HIF 1α factor [129,130]. Furthermore, Sha et al. proposed that OS9 serves as a substrate for the Sel1L/Hrd1 complex [131]. It has to be stressed that regulation between OS9 and Sel1L in cancer cells needs further investigation. An ATP-dependent process of extracting the ubiquitinated proteins from the ER membrane is mediated by the p97 ATPase [132]. Inhibition of the p97 ATPase is related to increased apoptosis of cancer cells [133,134,135]. In addition, increased expression of p97 ATPase in B cell lymphomas has been reported; however, ERS was not activated [133]. In contrast, primary lung adenocarcinoma patients expressed lower levels of p97 ATPase, which induced ERS [136].

Recent evidence suggests that ERS is involved in the regulation of resistance to chemotherapy either independent or dependent on P-gp [137,138,139,140,141]. Cagnetta et al. reported that P-gp inhibitors induced ERS and ERS blockage strongly reduced the cytotoxic effect of the treatment in leukemic cells [137]. The mechanism underlying the induction of ERS by P-gp inhibition is not completely clear. However, the fact that insulin resistance promotes PERK mediated ERS, expression of Bcl-2 and P-gp in human hepatocarcinoma cells [138] indicated on strong relations between these ESR and P-gp function. In addition, expression of PERK in ERS resistant cells was increased and resulted in Nrf2 dependent transcription of the MRP1 gene. The ERS and chemotherapy resistance were reversed by disrupting the PERK/Nrf2 axis [141]. Interestingly, the expression of the other ERS sensors IRE1α and ATF6 were not altered in resistant cells [140]. In contrast, downregulation of the ATF6 pathway promoted cell death and reversed the resistance of the dormant tumour cells to rapamycin and glioblastoma cells to radiation [142,143]. The transport activity of P-gp is considered to be the underlying mechanism of imatinib resistance in chronic myeloid leukaemia [144]. Kusio Kobialka et al. showed that PERK/eIF2α phosphorylation was associated with chronic myeloid leukaemia progression and imatinib resistance. Furthermore, imatinib-mediated apoptosis downregulated PERK/eIF2α phosphorylation [145].

The C/EBP family of proteins is a group of transcription factors involved in regulation of cellular responses to ERS [146]. Riganti et al. investigated the role of C/EBP-β and CHOP members in establishing MDR. Transcription factor C/EBP-β can have a pro apoptotic effect, which is mediated by the natural dominant negative, truncated transcriptional repressor liver-enriched inhibitory protein LIP isoform. On the other hand, the liver-enriched transcriptional activator protein LAP isoform also promotes tumour progression by attenuating ERS-triggered cell death [147]. They determined that MDR cells do not express LIP, which undergoes ubiquitin-mediated degradation and failed to activate the pro apoptotic CHOP/caspase-3 pathway upon ERS or chemotherapy induction. They proposed that the lack of LIP results in two independent actions, particularly in upregulation of P-gp and attenuation of ERS triggered apoptosis [140].

**Figure 3:**
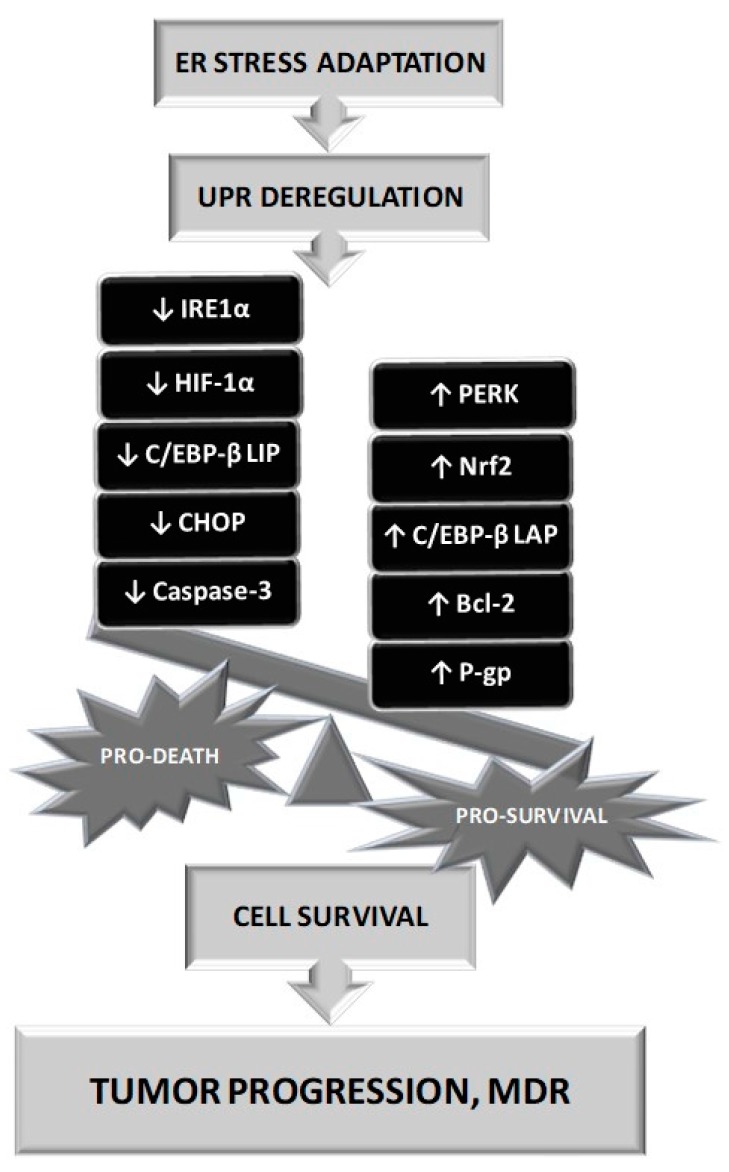
The role of Endoplasmic Reticulum Stress (ERS) in tumour progression and development of multidrug resistance. The adaptation to ERS leads to deregulation of UPR, activation of pro survival pathways, suppression of apoptotic pathways and overexpression of P-glycoprotein (P-gp) in malignant cells.

There also exists a gentle equilibrium between pro survival and pro death stimuli under ERS that is influenced by the severity of the stress and the duration of its presence. Under prolonged ERS, adaptation to this situation requires several steps and UPR deregulation may induce an imbalance of pro-survival mechanisms up to pro-death initiation and cells could escape from the death machinery induced by this situation (Figure 3).

## 6. Therapeutic Approaches

Firstly inhibition of P-gp with substances, which may be applied together with anticancer agents represents an important possibility to reduce P-gp antagonism against efficacy of cancer patients chemotherapy [148]. Recently, a new P-gp inhibitor tariquidar was developed as a product of rational drug design [149]. Tariquidar represents a high affinity, uncompetitive P-gp inhibitor that in contrast to verapamil, cyclosporine A and their analogues cannot be transported by P-gp [150]. This substance and its analogues are under intensive research to optimize the effective protocols for multidrug resistant malignancy treatment. For example, tariquidar in combination with doxorubicin, docetaxel, or vinorelbine in children and adolescents with recurrent or refractory solid tumours was tested clinically and a tolerable and biologically active dose of tariquidar was established [151].

Secondly targeting the UPR pathways in cancer cells may lead to a higher survival rate and reduced resistance of the cells to chemotherapy. Furthermore, the expression of ERS-mediated transcription factors may prove useful as prognosis indicators. The ERS inducers thapsigargin, tunicamycin and brefeldin A accelerated tumour growth in mice and human cancer cells [141,152]. In contrast, tunicamycin reduced tumour growth either in triple negative or double negative breast cancer models [153]. Cell treated by specific inhibitors of the UPR downstream mediators or mice malignant cells lacking IRE1α, XBP-1, PERK, or ATF6 are more sensitive to hypoxia, with higher production of ROS, lower angiogenesis, delayed tumour progression and metastasis [139,154,155,156,157,158,159,160,161,162,163]. Several inhibitors of IRE1α were identified in the last decade [159,164,165,166,167,168,169,170]. Inhibition of the IRE1α/XBP-1 pathway by toyocamycin, salicylaldehyde analogues and STF 083010 induced apoptosis in various tumours [167,168,169]. In addition, two new IRE1α inhibitors (4μ8C and KIRAs) that act allosterically were developed [159,164]. Two small molecules (GSK2656157 and GSK2606414) as selective PERK inhibitors were proposed [154,156]. Morrow et al. showed that pegylated-human-arginase I induced lymphoblastic T-cell leukaemia apoptosis by inhibiting the phosphorylation of eIF2α [171]. Furthermore, chemical chaperones, tauroursodeoxycholic acid and 4-phenyl butyric acid, which alleviate ERS, reduced tumour growth and tumorigenesis [152,170]. Proteasome inhibitors, bortezomib and toyocamycin, were also reported to suppress the PERK and IRE1α pathways, leading to increased apoptosis of cancer cells [160,172]. The cytotoxic effect of the proteasome inhibitors was promoted in combination with the p97 ATPase inhibitor eeyarestatin I [173,174]. In addition, IRE1α and ATF6 silencing promoted the apoptotic effect of rhabdovirus-mediated oncolysis [175]. On the contrary, valproic acid, a histone deacetylase inhibitor that is another promising chemotherapeutic agent, induced UPR by upregulating the expression of BiP, CHOP and Sel1L in glioma stem cells [176] and the chemical chaperone 4-phenyl butyric acid, an ERS inhibitor, reduced the cytotoxic effect of chemotherapeutics [137]. Furthermore, lower expression of Sel1L in glioma stem cells with the SNP rs12435998 genotype was related to enhanced sensitivity to valproic acid [128].

Several studies recently investigated the link between ERS and the resistance of cells to chemotherapy (Table S1 in Supplementary Files). Both activation and inhibition of ERS sensors were proposed to regulate the development of MDR. HIV protease inhibitors, which blocked ERS, decreased the transport activity of P-gp resulting in accumulation of berberine in macrophages [177]. Similarly, reduced tumour growth and restored chemosensitivity in resistant tumours were observed after PERK silencing [141,145]. On the other hand, the increased activation of PERK was associated with upregulation of P-glycoprotein and resistance to adriamycin in hepatocarcinoma cells [178]. Furthermore, the inhibition of IRE1α significantly improved the efficacy of oncolytic virus therapy in resistant tumour models [175].

Several studies recently investigated the link between ERS and the resistance of cells to chemotherapy (Table S1 in Supplementary Files). Both activation and inhibition of ERS sensors were proposed to regulate the development of MDR. HIV protease inhibitors, which blocked ERS, decreased the transport activity of P-gp resulting in accumulation of berberine in macrophages [177]. Similarly, reduced tumour growth and restored chemosensitivity in resistant tumours were observed after PERK silencing [141,145]. On the other hand, the increased activation of PERK was associated with upregulation of P-glycoprotein and resistance to adriamycin in hepatocarcinoma cells [178]. Furthermore, the inhibition of IRE1α significantly improved the efficacy of oncolytic virus therapy in resistant tumour models [175]. 

Chakravarty et al. [179] have investigated the effect of nelfinavir, an HIV protease inhibitor (also known as P-gp substrate [180]) on doxorubicin toxicity in an MDR breast cancer cell line. They reported that single exposure to nelfinavir transiently induced P-gp levels; however, multiple treatments with nelfinavir inhibited both P-gp expression and efflux activity together with activation of the pro apoptotic PERK/ATF4/CHOP pathway. Another study described the inhibition of XBP-1 in multiple myeloma cells, which induced bortezomib resistance [181]. Furthermore, rat C6 glioma cells developed resistance to emodin, an antitumor agent, which was related to overexpression of MDR genes and reduced ERS [182]. Thapsigargin, an ERS inducer, decreased the expression of P-gp in leukaemia cells and treatment with cyclosporine A, a P-gp inhibitor, increased expression of IRE1α [137,183]. Similarly, increased chemosensitivity of melanoma stem-like cells was associated with induction of ERS [184]. Finally, Song et al. reported that ERS reduced drug resistance in breast cancer; however, the transport activity of P-gp was not altered [185]. 

Tunicamycin, blocks protein *N*-glycosylation and thus leads to the accumulation of unfolded proteins and the activation of ERS [186,187]. It was reported that inhibition of *N*-glycosylation, a major posttranslational modification of P-gp, by tunicamycin caused rapid ubiquitination and proteasome-dependent degradation of P-gp [188,189]. Furthermore, Kramer et al. reported that tunicamycin reversed P-gp mediated MDR [190]. In contrast, our studies showed that tunicamycin failed to reverse the efflux activity of P-gp in leukaemia cells, suggesting that unglycosylated P-gp has the ability to escape from the ERAD system and become functionally integrated into the plasma membrane [191,192,193]. This is in line with the Riganti et al. study, where tunicamycin did not alter the efflux activity or levels of P-gp, MRP1 or MRP2 [140]. Interestingly, unglycosylated P-gp that was found in the membrane and exerted P-gp efflux activity as measured by a calcein retention assay (after treatment with tunicamycin [193]) was ubiquitinated [191]. Thus, after ubiquitination, P-gp continued to mature instead of being degraded in proteasomes. Ubiquitin is a small polypeptide (76 amino acids, M_r_ 8500 kDa) that can conjugate with several proteins via formation of isopeptide bond between ubiquitin C-terminal glycine and lysine in target protein. Ubiquitin contains seven lysines (K6, K11, K27, K29, K33, K48 and K63) and any of them could form isopeptide bond with C-terminal glycine of another ubiquitin molecule to form polyubiquitin chain [194]. Therefore, ubiquitination produces either monoubiquitinated or polyubiquitinated proteins. Protein ubiquitination leads to increase of protein molecular weight reflecting the number of ubiquitins linked in polyubiquitin chains attached to protein [195]. Monoubiquitination seems to be followed by chromatin regulation, protein sorting and trafficking, whereas polyubiquitination is rather associated with protein degradation in proteasome [196]. In previous paper, we observed larger P-gp ubiquitination after treatment of P-gp positive variants of mouse leukaemia cell line L1210 with tunicamycin. This ubiquitination was associated with only small elevation in P-gp molecular weight, which excludes massive polyubiquitination [191]. Ubiquitinated and active P-gp was still localized in plasma membrane [193]. Beside number of ubiquitins in polyubiquitin chains also structural feature, i.e. which from seven lysines was used for isopeptide bond formation is important for final direction of ubiquitin signalling. For example, proteasomal degradation is typical consequence of protein polyubiquitination through lysine in position 48 on ubiquitin molecule (conventional chain). However, similarly abundant polyubiquitination utilizing K63 leads rather to kinase activation, DNA- repair and vesicle trafficking [197]. Taken together, the destiny of ubiquitinated proteins, after blockage of *N*-glycosylation and overall quality control in ER by tunicamycin, depends on the number of bound ubiquitins in the chain and, in the case of polyubiquitination, on special structural features of the polyubiquitin chain [191]. 

## 7. Conclusions

Cancer cells are able to take advantage of cell programs, which serve to maintain physiological function of the cells under stress conditions. Persistent ERS, due to different pathological impulses including hypoxia and glucose shortage in the tumour environment, or the presence of ER stressors, should result in cell death. However, cancer cells are able to stay in an extremely prolonged early pro survival response to ERS. Moreover, cancer cells can induce expression of P-gp and use its efflux activity for removal of xenobiotics to survive the cytotoxic effect of chemotherapy. The mechanism of how malignant cells adapt to ERS and overcome the UPR cell death program is crucial for understanding the progression and resistance of tumours to therapy. One of the possibilities is that adaptation to ERS by cancer cells may lead to overexpression and induction of P-gp and thus contribute to production of an MDR phenotype. It seems that increases of PERK activity, an ERS sensor and loss of C/EBP LIP in resistant cells results in overexpression of P-gp. Furthermore, modification of the ERAD system can be observed in the case of unglycosylated P-gp (due to *N*-glycosylation blockage by tunicamycin [193]), which after ubiquitination could escape from the proteasomal degradation cascade and in ubiquitinated form is integrated into the plasma membrane and maintains its function [191]. Therefore, targeting the UPR pathways in cancer cells may lead to disturbed P-gp function and reverse resistance of the cells to chemotherapy. These facts suggest a new role of ERS sensors and ERAD components as therapeutic targets in the treatment of resistant tumours. In addition, mutation of the ERS sensor genes, particularly the missense mutations enriched in PERK, have been proposed to be responsible for the changes in UPR [198,199]. However, mutations differ among various types of cancers [200]; thus, characterization of tumour specific mutations and the impact of ERS adaptation in the relationship to P-gp dependent MDR is needed to verify the above hypotheses. 

## Supplementary Materials

The following are available online.

## Abbreviations

ATF6	activating transcription factor 6
ABC	ATP-binding cassette
BiP	immunoglobulin binding protein
CHOP	C/EBP homologous protein
eIF2α	eukaryotic initiation translation factor 2α
ER	endoplasmic reticulum
ERAD	ER associated protein degradation
ERS	ER stress
GA	golgi apparatus
GRP78	glucose regulated protein 78
HSP	heat-shock protein
iASPP	inhibitor of apoptosis-stimulating protein p53
IRE1α	inositol-requiring enzyme 1α
keap1	Kelch-like ECH-associated protein 1
MDR	multidrug resistance
mTOR	mechanistic target of rapamycin
Nrf2	nuclear factor-erythroid 2-related factor 2
OS9	osteosarcoma amplified 9
PERK	pancreatic ER kinase
P-gp	P-glycoprotein
ROS	reactive oxygen species
S1P and S2P	site 1 and site 2 proteases
UGGT	UDP-glucose:glycoprotein glycosyltransferase
URP	unfolded protein response
VEGF	vascular endothelial growth factor
XBP1	X-box-binding protein
XTP3	lectin of ER
XTP3-B	transactivated protein or ER lectin
