# Supplementary material for: Interplay between P-Glycoprotein Expression and Resistance to Endoplasmic Reticulum Stressors
Source: Molecules. 2018 Feb 6;23(2):337. doi: 10.3390/molecules23020337 (PMC6017601; doi:10.3390/molecules23020337)
Supplement: Supplementary file 1 [file molecules-23-00337-s001.pdf]

Hano et al.:

Interplay between P-glycoprotein expression and resistance to endoplasmic reticulum stressors.

Suppplementary files.

Table 1: Comparison of ER stress-mediated responses to chemotherapy in resistant tumors

**Treatment**		**Cell line**	**ER stress related mechanism**	**Cytotoxic effect**	
Emodin	PKC inhibitor	Rat C6 glioma cells	↑BiP and CHOP expression	↑P-gp expression<br>↑resistance	[1]
Thapsigargin	SERCA inhibitor	Mouse L1210 leukemia cells	↓Calnexin	↓P-gp expression	[2]
Rhabdovirus	Oncolytic anticancer virus therapy	Human U373 glioblastoma cells	IRE1α knockdown/inhibition (SA)<br>XBP-1 knockdown	↑cytotoxicity<br>↓tumor growth	[3]
		Various cell lines	IRE1α and ATF6 knockdown		
		Mice OVCAR-4 ovarian carcinoma	IRE1α inhibition (SA)		
		Glioblastoma patient samples, mouse EMT6 breast cancer cells	IRE1α inhibition (SA)		
Imatinib	BCR-ABL tyrosine kinase inhibitor	Mouse 32Dc13 and human K562 chronic myeloid leukemia cells	↓PERK/eIF2α phosphorylation	↓resistance	[4]
Bortezomib	Proteasome inhibitor	Human KMS11 multiple myeloma cells	IRE1α and XBP-1 knockdown	↑resistance	[5]
S1	Pan-BH3 mimetic	Human MCF-7 breast adenocarcinoma cells	↑IRE1α expression	no influence on P-gp expression<br>↑cytotoxicity	[6]
Amprenavir<br>Ritonavir	HIV protease inhibitors	Mouse RAW 264.7 macrophages	↑CHOP, XBP-1, ATF4 expression	↓P-gp efflux activity<br>↓berberine resistance	[7]<br>[8]
APO866<br>+ cyclosporine A	NAMPT inhibitor<br>+ P-gp inhibitor	Human B-CLL leukemia cells	↑IRE1α, CHOP, BiP expression	P-gp inhibition<br>↑cytotoxicity	[9]
CAPE	Anticancer drug	Human MV3 melanoma cells	↑IRE1α, PERK phosphorylation	ABCB5 inhibition<br>↑cytotoxicity	[10]
Doxorubicin	Anticancer drug	Human HepG2/IR hepatoma cells	↑BiP and PERK expression	↑P-gp expression<br>↑resistance	[9]
Nelfinavir	HIV protease inhibitor	Human MCF-7 breast adenocarcinoma cells	↑PERK/eIF2α phosphorylation<br>↑BiP, CHOP expression	↓P-gp expression<br>↓P-gp efflux activity<br>↓doxorubicin resistance	[11]
Thapsigargin,<br>Tunicamycin<br>Brefeldin A	SERCA inhibitor<br>N-glycosylation inhibitor<br>Golgi-membrane translocation inhibitor	Human HT29 colon cancer cells	↑PERK/Nrf2 expression	↑MRP1 expression<br>↑doxorubicin resistance	[12]

ER, endoplasmatic reticulum; P-gp, P-glycoprotein; MRP, multidrug resistance protein; GRP78, 78-kDa glucose-regulated protein; BiP, immunoglobulin binding protein; IRE1α, inositol-requiring enzyme 1α; XBP, X-box-binding protein; PERK, pancreatic endoplasmic reticulum kinase; eIF2α , eukaryotic initiation translation factor 2α; ATF, activating transcription factor; CHOP, C/EBP homologous protein; Nrf2, nuclear factor-erythroid 2-related factor 2; SA, salicylaldehyde analogs; CAPE, Caffeic acid phenethyl ester; PKC, proteinkinase C; SERCA, sarco/endoplasmic reticulum Ca^{2+}-ATPase; HIV, human immunodeficiency virus; NAMPT, nicotinamide phosphoribosyltransferase

References

1. Kuo, T.C.; Yang, J.S.; Lin, M.W.; Hsu, S.C.; Lin, J.J.; Lin, H.J.; Hsia, T.C.; Liao, C.L.; Yang, M.D.; Fan, M.J., *et al.* Emodin has cytotoxic and protective effects in rat c6 glioma cells: Roles of mdr1a and nuclear factor kappab in cell survival. *J Pharmacol Exp Ther* **2009**, *330*, 736-744.
2. Seres, M.; Ditte, P.; Breier, A.; Sulova, Z. Effect of thapsigargin on p-glycoprotein-negative and p-glycoprotein-positive l1210 mouse leukaemia cells. *Gen Physiol Biophys* **2010**, *29*, 396-401.
3. Mahoney, D.J.; Lefebvre, C.; Allan, K.; Brun, J.; Sanaei, C.A.; Baird, S.; Pearce, N.; Gronberg, S.; Wilson, B.; Prakesh, M., *et al.* Virus-tumor interactome screen reveals er stress response can reprogram resistant cancers for oncolytic virus-triggered caspase-2 cell death. *Cancer Cell* **2011**, *20*, 443-456.
4. Kusio-Kobialka, M.; Podszywalow-Bartnicka, P.; Peidis, P.; Glodkowska-Mrowka, E.; Wolanin, K.; Leszak, G.; Seferynska, I.; Stoklosa, T.; Koromilas, A.E.; Piwocka, K. The perk-eif2alpha phosphorylation arm is a pro-survival pathway of bcr-abl signaling and confers resistance to imatinib treatment in chronic myeloid leukemia cells. *Cell Cycle* **2012**, *11*, 4069-4078.
5. Leung-Hagesteijn, C.; Erdmann, N.; Cheung, G.; Keats, J.J.; Stewart, A.K.; Reece, D.E.; Chung, K.C.; Tiedemann, R.E. Xbp1s-negative tumor b cells and pre-plasmablasts mediate therapeutic proteasome inhibitor resistance in multiple myeloma. *Cancer Cell* **2013**, *24*, 289-304.
6. Song, T.; Liang, F.; Zhang, Z.; Liu, Y.; Sheng, H.; Xie, M. S1 kills mcf-7/adr cells more than mcf-7 cells: A protective mechanism of endoplasmic reticulum stress. *Biomed Pharmacother* **2013**, *67*, 731-736.
7. Atkins, C.; Liu, Q.; Minthorn, E.; Zhang, S.Y.; Figueroa, D.J.; Moss, K.; Stanley, T.B.; Sanders, B.; Goetz, A.; Gaul, N., *et al.* Characterization of a novel perk kinase inhibitor with antitumor and antiangiogenic activity. *Cancer Res* **2013**, *73*, 1993-2002.
8. Bobrovnikova-Marjon, E.; Grigoriadou, C.; Pytel, D.; Zhang, F.; Ye, J.; Koumenis, C.; Cavener, D.; Diehl, J.A. Perk promotes cancer cell proliferation and tumor growth by limiting oxidative DNA damage. *Oncogene* **2010**, *29*, 3881-3895.
9. Cagnetta, A.; Caffa, I.; Acharya, C.; Soncini, D.; Acharya, P.; Adamia, S.; Pierri, I.; Bergamaschi, M.; Garuti, A.; Fraternali, G., *et al.* Apo866 increases antitumor activity of cyclosporin-a by inducing mitochondrial and endoplasmic reticulum stress in leukemia cells. *Clin Cancer Res* **2015**, *21*, 3934-3945.
10. El-Khattouti, A.; Sheehan, N.T.; Monico, J.; Drummond, H.A.; Haikel, Y.; Brodell, R.T.; Megahed, M.; Hassan, M. Cd133(+) melanoma subpopulation acquired resistance to caffeic acid phenethyl ester-induced apoptosis is attributed to the elevated expression of abcb5: Significance for melanoma treatment. *Cancer Lett* **2015**, *357*, 83-104.
11. Chakravarty, G.; Mathur, A.; Mallade, P.; Gerlach, S.; Willis, J.; Datta, A.; Srivastav, S.; Abdel-Mageed, A.B.; Mondal, D. Nelfinavir targets multiple drug resistance mechanisms to increase the efficacy of doxorubicin in mcf-7/dox breast cancer cells. *Biochimie* **2016**, *124*, 53-64.
12. Salaroglio, I.C.; Panada, E.; Moiso, E.; Buondonno, I.; Provero, P.; Rubinstein, M.; Kopecka, J.; Riganti, C. Perk induces resistance to cell death elicited by endoplasmic reticulum stress and chemotherapy. *Mol Cancer* **2017**, *16*, 91.
